# Coupled Effects of Elevated Water Pressure and Limestone Powder on Thaumasite Sulfate Attack in Cement Mortar

**DOI:** 10.3390/ma19091858

**Published:** 2026-04-30

**Authors:** Hao Li, Tao Han, Yingfeng Tan, Weihao Yang

**Affiliations:** State Key Laboratory of Intelligent Construction and Healthy Operation and Maintenance of Deep Underground Engineering, School of Mechanics and Civil Engineering, China University of Mining and Technology, Xuzhou 221116, China

**Keywords:** thaumasite sulfate attack, water pressure, cement mortar, coupled mechanisms, microstructural evolution

## Abstract

Thaumasite sulfate attack (TSA) under elevated water pressure has important implications for the durability of deep underground concrete structures, yet the deterioration process and the coupled effect of water pressure and carbonate supply remain insufficiently understood. In this study, laboratory pressurized sulfate exposure tests were conducted to investigate the evolution of macroscopic performance and microstructure of cement mortars with different limestone powder contents (0%, 15%, and 30%) under water pressures of 0, 2.5, and 5.0 MPa. The results show that elevated water pressure promotes sulfate ingress into the mortar and accelerates later-stage strength loss; this interpretation is supported by the depth-dependent distribution of soluble SO_4_^2−^ measured in mortars without limestone powder. Two-way ANOVA indicates that both water pressure and limestone powder content have significant effects on compressive strength, and their interaction becomes statistically significant at 120 d. XRD, FT-IR, and SEM/EDS results show that, under elevated water pressure and high limestone powder content, the corrosion products gradually evolve from gypsum-related products to ettringite- and thaumasite-related products, with a certain spatial differentiation. Specifically, the gray–white, mud-like surface products are consistent with thaumasite-rich assemblages, whereas the needle- and column-like crystals in the interior are consistent with ettringite-rich assemblages. Overall, elevated water pressure mainly promotes sulfate transport, while limestone powder mainly increases carbonate availability. These two factors may jointly intensify TSA deterioration in mortar through a pathway involving transport enhancement, carbonate supply, corrosion product evolution, and aggravated macroscopic damage. This study provides a reference for understanding the sulfate deterioration mechanism of limestone powder-containing cement-based materials in deep underground environments under elevated water pressure.

## 1. Introduction

Thaumasite sulfate attack (TSA) is a severe form of sulfate-induced deterioration in cement-based materials, characterized by the transformation of cementitious phases into non-cementitious products, resulting in significant strength degradation and structural instability. Since its first identification in 1965 [1], TSA has been widely reported in hydraulic structures and underground engineering, posing a serious threat to the durability and serviceability of concrete infrastructures. Recent reviews have further summarized the formation pathways, influencing factors, and mitigation strategies of TSA in cement-based materials, emphasizing that thaumasite formation is closely related to the simultaneous availability of sulfate, carbonate, calcium, and silicate sources, as well as environmental conditions such as temperature and moisture supply [2,3]. In addition, recent studies on sulfate attack have highlighted that the deterioration of cement-based materials should be understood as a coupled process involving ion transport, chemical reactions, crystallization or precipitation of corrosion products, and the subsequent degradation of mechanical properties [4,5].

With the increasing demand for deep underground resource exploitation, the depth of mine shafts has continuously increased, leading to more severe service conditions for concrete structures [6,7,8,9]. In deep underground environments, concrete shaft linings are subjected to the combined effects of high ground stress, sulfate-rich groundwater, and elevated water pressure, which significantly accelerate deterioration processes [10,11,12,13,14]. Field investigations have reported severe damage in shaft structures exposed to such environments. For example, in Laiwu Mine (Shandong, China), serious corrosion of the shaft lining occurred within less than 5 years of service, with material degradation reaching depths of 5–10 cm, allowing manual disintegration of the concrete [15]. Similarly, in the Mataihao Coal Mine (Inner Mongolia, China), rapid deterioration, including peeling, pulverization, and shedding, was observed, with corrosion depths of 8–15 cm under groundwater sulfate concentrations exceeding 1500 mg/L [16]. Similar TSA-related damage has also been reported in buried concrete structures, such as highway bridges and tunnels [17,18,19,20,21]. These observations indicate that TSA under high water pressure is a critical durability issue in deep underground engineering. Under such conditions, sulfate ingress driven by water pressure may become an important factor governing deterioration. The penetration of sulfate ions into the concrete matrix, coupled with sustained water pressure, promotes ion transport, accelerates internal reactions, and leads to progressive damage from the surface to the interior. Despite the severe engineering implications, the mechanisms of TSA under elevated water pressure remain insufficiently understood.

Extensive studies have been conducted to investigate the mechanisms and influencing factors of TSA in cement-based materials. Recent reviews have indicated that TSA development is affected by multiple factors, including temperature, sulfate concentration, and the availability of carbonate ions [2,22,23,24]. Among these factors, carbonate availability is particularly important because thaumasite formation requires the participation of carbonate together with sulfate, calcium, and silicate species. Therefore, limestone powder, which is increasingly used as a partial cement replacement or filler, may increase the risk of TSA by providing an additional carbonate source under suitable exposure conditions [25,26,27,28]. Recent experimental studies on limestone powder-containing cementitious systems have also shown that sulfate attack behavior is strongly dependent on temperature and exposure conditions, and thaumasite-related deterioration is more likely to occur under low-temperature sulfate environments [26]. However, most existing studies have been performed under normal or low water pressure conditions (typically below 3 MPa), where ion transport is primarily governed by diffusion. Recent experimental evidence on TSA and sulfate-exposed mortars still mainly concerns conventional exposure conditions, while systematic studies under elevated water pressure remain scarce [29,30]. Under such conditions, gypsum and ettringite are generally considered the main corrosion products, and the formation of thaumasite is relatively limited.

Although recent studies have improved the understanding of TSA mechanisms, sulfate attack testing methods, transport–reaction–damage coupling, and the influence of water pressure or water penetration on concrete damage and transport behavior, the role of elevated water pressure in TSA remains insufficiently clarified [4,12,13,25,26]. In deep underground environments, water pressure can reach several megapascals, which may promote the ingress of sulfate solution and influence transport and reaction processes within the cement matrix. However, the coupling between water pressure-assisted sulfate supply and limestone powder-derived carbonate availability has rarely been systematically investigated. Moreover, the influence of elevated water pressure on corrosion product evolution, spatial distribution, and the associated deterioration mechanism remains unclear.

Therefore, this study aims to investigate the degradation behavior of cement mortar subjected to TSA under the combined effects of elevated water pressure and limestone powder, with particular attention to their coupling in sulfate transport, corrosion product formation, and macroscopic performance degradation. Specifically, this study attempts to answer the following questions: whether elevated water pressure promotes the migration of sulfate solution into mortar; whether the carbonate source provided by limestone powder changes the formation characteristics of TSA-related corrosion products; and whether the simultaneous presence of water pressure-driven sulfate supply and limestone powder-derived carbonate supply jointly affects corrosion product evolution, spatial distribution, and strength degradation of mortar. To this end, accelerated sulfate exposure tests were conducted on mortar specimens with different limestone powder contents under controlled water pressure environments. Compressive strength testing, depth-dependent soluble SO_4_^2−^ content measurements, and microstructural characterization using XRD, FT-IR, and SEM/EDS were combined to analyze the coupled effects of elevated water pressure and limestone powder on TSA deterioration.

## 2. Experimental Procedure

To investigate the coupled effects of elevated water pressure and limestone powder on TSA in cement mortar, a two-factor accelerated corrosion test was designed with different limestone powder contents and water pressure levels. This design was used not only to evaluate the individual effects of water pressure and limestone powder content on mortar deterioration, but also to analyze their interaction in sulfate transport, corrosion product formation, and macroscopic performance degradation. By characterizing compressive strength, macroscopic deterioration morphology, corrosion products, and microstructural features during exposure, the deterioration evolution of cement mortar under elevated water pressure was analyzed. For this purpose, X-ray diffraction (XRD), Fourier-transform infrared spectroscopy (FT-IR), and scanning electron microscopy with energy-dispersive spectroscopy (SEM/EDS) were used to analyze the corrosion products, while supplementary soluble SO_4_^2−^ content measurements were used to support the interpretation of sulfate ingress characteristics. This section sequentially describes the raw materials and mixture proportions, specimen preparation and curing, elevated-water-pressure exposure system and test design, sample collection and preparation, testing methods and data processing.

### 2.1. Materials and Mixture Proportions

To simulate the deterioration process of cement-based materials under the combined action of sulfate ions and carbonate ions in underground environments, mortar specimens incorporating different proportions of limestone powder were prepared. The introduction of limestone powder provided an internal carbonate source for thaumasite formation and was also used to examine the influence of carbonate availability on TSA development [26,28].

P·O 42.5 Portland cement supplied by Xuzhou-Zhonglian Cement Co., Ltd. (Xuzhou, China), quartz sand (particle size 0.2–2 mm) supplied by Lianyungang-Dingmai Quartz Products Co., Ltd. (Lianyungang, China), limestone powder (200 mesh, purity > 98%, whiteness > 95%) produced by Baoxing-Zhangzheng Powder Co., Ltd. (Ya’an, China), and tap water were used to prepare the mortar specimens. The chemical composition of the cement is presented in Table 1. The sulfate corrosion solution was prepared using analytical-grade anhydrous sodium sulfate with a purity greater than 99%. According to the limestone powder content, the specimens were divided into three groups: 0%, 15%, and 30%, corresponding to groups B1, B2, and B3, respectively. The mortar mixture proportions are shown in Table 2.

### 2.2. Specimen Preparation and Curing

Mortar specimens were prepared according to the mixture proportions shown in Table 2. Mortar specimens with dimensions of 70.7 mm × 70.7 mm × 70.7 mm were cast in steel molds. After casting, the specimens were stored in a laboratory environment at (20 ± 5) °C for 72 h before demolding. Subsequently, all specimens were cured under standard conditions at (20 ± 2) °C and a relative humidity of 95% for 28 days. After curing, the specimens were grouped according to the subsequent test scheme and used for elevated water pressure sulfate exposure tests.

### 2.3. Test Procedure

#### 2.3.1. Elevated Water Pressure Corrosion Test System

**Figure 1:**
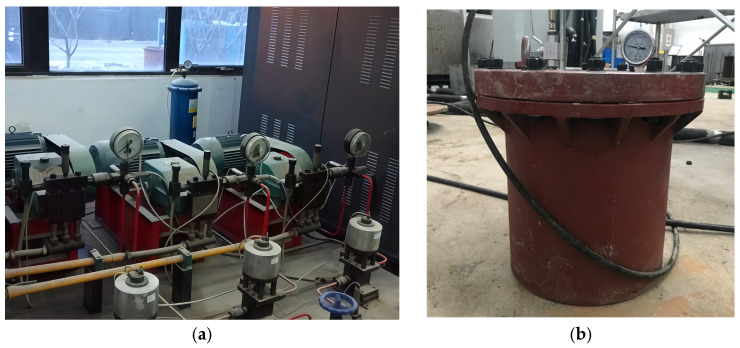
Experimental system: (**a**) servo-controlled pressure stabilization system; (**b**) the high-pressure sulfate corrosion reactor.

To simulate the service conditions of cement mortar under the combined action of sulfate solution and elevated water pressure in deep underground environments, a servo-controlled pressure stabilization system was used for the test, as shown in Figure 1a. The system consists of independent high-pressure oil and water circuits and can realize stable loading and continuous control of water pressure. The maximum loading pressure of the system is 80 MPa, with an accuracy of 0.1 MPa, which satisfies the requirements of the exposure tests at different water pressure levels in this study.

To provide a pressurized corrosion environment, a cylindrical pressure vessel made of 45 steel (code name U20452, yield strength 355 MPa) was used, as shown in Figure 1b. The vessel has an inner radius of 0.2 m and a height of 0.4 m. The wall thickness was set to 10 mm, corresponding to approximately 3.57 times the theoretical value, to ensure adequate safety under high-pressure conditions.

#### 2.3.2. Description of the Water Pressure Action Mode

In this study, the mortar specimens were directly immersed in sodium sulfate solution and subjected to a constant external water pressure to simulate the long-term corrosion of cement-based materials by sulfate-rich groundwater under elevated water pressure in deep underground environments. Compared with atmospheric immersion, the external solution under elevated water pressure is more likely to migrate into the specimen along surface pores and pre-existing defects, thereby increasing the contact opportunities between the aggressive medium and internal hydration products and accelerating the development of corrosion reactions.

Therefore, the pressurized exposure conditions in this study are mainly used to characterize the promoting effect of water pressure on sulfate ingress and damage evolution, rather than simply representing hydrostatic saturation of the material. In combination with mortar specimens containing different limestone powder contents, this design further enables analysis of the combined action of water pressure-driven transport and carbonate availability during TSA deterioration.

#### 2.3.3. Test Design and Grouping

A two-factor experimental design was adopted in this study, with limestone powder content and water pressure level serving as the main variables. The limestone powder contents were set at 0%, 15% and 30%, corresponding to groups B1, B2 and B3, respectively. The water pressure levels were set at 0 MPa, 2.5 MPa, and 5.0 MPa, where 0 MPa represents atmospheric immersion and 2.5 MPa and 5.0 MPa represent elevated water pressure exposure. All specimens were immersed in a 10 wt% Na_2_SO_4_ solution to simulate a sulfate-rich groundwater environment. The purpose of this two-factor design was not only to compare the effects of a single water pressure level or a single limestone powder content on deterioration, but more importantly to evaluate their coupling in sulfate transport, corrosion product formation, and strength degradation.

According to the above variable combinations, a total of nine test conditions were established. The specimen group IDs and corresponding test conditions are listed in Table 3. For each group ID, the first part indicates the limestone powder content group, while the second part indicates the water pressure level. For example, B2-3 denotes specimens with 15% limestone powder content exposed to a water pressure of 5.0 MPa. The exposure ages for all groups were set as 30 d, 60 d, 90 d, and 120 d.

#### 2.3.4. Exposure Conditions and Test Procedure

After 28 d of standard curing, the mortar specimens in each group were placed in a 10 wt% Na_2_SO_4_ solution for corrosion exposure. The test temperature was the natural room temperature in autumn and winter, approximately (12 ± 5) °C, and the volume ratio of solution to specimens was approximately 3:1. To maintain the stability of the corrosion environment, the sulfate solution was renewed every 15 d during the test.

The specimens in the atmospheric immersion group were placed in sealed plastic containers. Their exposure temperature, solution-to-specimen volume ratio, solution renewal interval, and sampling ages were kept consistent with those of the elevated water pressure groups. For the high-pressure immersion groups, the specimens were placed in the high-pressure reactor and continuously exposed to sodium sulfate solution under the corresponding applied water pressure. During the test, the pressure state was monitored and adjusted in real time using the servo-controlled pressure stabilization system to ensure that each group of specimens remained under the preset pressure condition.

At the specified exposure ages, the corresponding specimens were removed for subsequent testing and analysis. After sampling, the specimen surfaces were first cleaned and labeled, and their apparent deterioration morphology was recorded. The specimens were then used for compressive strength testing, microstructural characterization, or soluble SO_4_^2−^ content testing according to the testing purpose. Throughout the test, all exposure conditions except for the preset water pressure were kept as consistent as possible among the different groups to ensure comparability of the results.

In particular, to support the analysis of sulfate ingress characteristics under different water pressure conditions, additional one-dimensional corrosion specimens were prepared for soluble SO_4_^2−^ content measurements. Their sampling and sample preparation methods are described in Section 2.4.2.

### 2.4. Sampling and Sample Preparation

#### 2.4.1. Sampling and Preparation of Samples for Microstructural Analysis

After reaching the specified exposure age, one representative sample was selected from the corresponding test group for XRD, FT-IR, or SEM/EDS microstructural analysis. To compare differences in the composition and morphology of corrosion products in different regions, surface and internal samples were collected separately. Surface samples were mainly taken from gray–white loose corrosion products near the exposed surface. Internal samples were collected from dense regions away from the outer surface after splitting the specimen, so as to minimize the influence of surface corrosion products on the internal analysis results. For specimens used to compare surface and internal corrosion products, the surface and internal samples were taken from different regions of the same specimen to improve the correspondence and comparability among the different test results. It should be noted that, except for compressive strength tests, the XRD, FT-IR, and SEM/EDS tests in this study were conducted on representative samples and were mainly used for qualitative or semi-quantitative analysis rather than statistical replicate testing.

**Figure 2:**
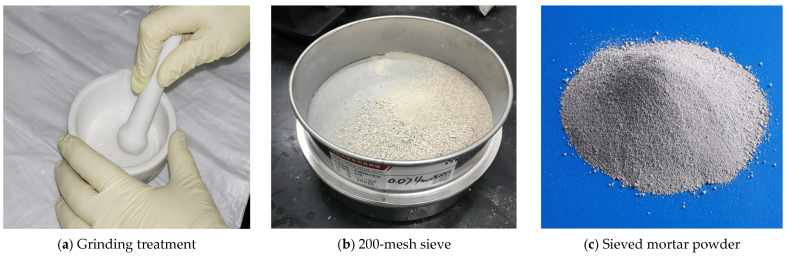
Pretreatment procedure of cement mortar powder for microscopic testing.

After sampling, the hardened mortar samples to be tested was dried to constant mass in a vacuum oven at 50 °C. The dried samples were placed in sealed bags, cooled to room temperature, then crushed into small particles, and thoroughly ground in a mortar. Finally, the powders were passed through a 200-mesh (approximately 80 μm) square-hole sieve. The pretreatment procedure is shown in Figure 2. The sieved powders were labeled and sealed for subsequent XRD and FT-IR tests.

**Figure 3:**
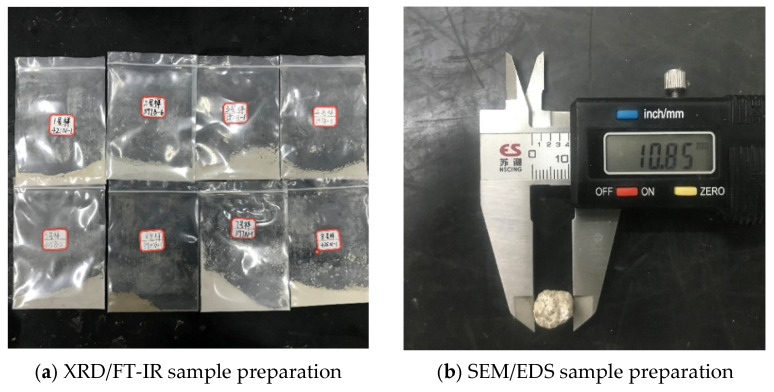
Sample preparation for microscopic characterization tests.

Samples for SEM/EDS testing were cut from the specimen surface or the internal cross-section, with an observation area of approximately 1 cm^2^. During sampling, the original morphology of the corrosion products was preserved as much as possible to avoid excessive damage to the surface structure. After drying, the observation surfaces were sputter-coated with gold to improve electrical conductivity and reduce charge accumulation during testing. The sample preparation for microscopic characterization tests is shown in Figure 3.

#### 2.4.2. Sampling and Preparation of Samples for Soluble SO_4_^2−^ Content Testing

To support the analysis of sulfate ingress characteristics in mortar under different water pressure conditions, specimens from groups B1-1, B1-2, and B1-3 without limestone powder were selected for supplementary soluble SO_4_^2−^ content measurements. This test was used as a supplementary analysis. Because it was mainly intended to support the interpretation of sulfate ingress characteristics under different water pressure conditions, only one representative cubic specimen with dimensions of 70.7 mm × 70.7 mm × 70.7 mm was selected from each group, and the soluble SO_4_^2−^ content was measured only at 60 d of corrosion exposure to characterize the preliminary distribution of sulfate within the mortar under different water pressure conditions. Except that the exposure mode for the specimens used for SO_4_^2−^ content measurement was adjusted to one-dimensional sulfate exposure, the remaining corrosion conditions were kept consistent with the corresponding test groups, thereby ensuring comparability of the results under different water pressure conditions. Since only one representative specimen was used for each group in this test, the results were used mainly as supplementary evidence supporting the promoting effect of water pressure on sulfate ingress and were not used for statistical analysis.

To restrict the corrosion process to a single direction, four opposing side surfaces among the six surfaces of each cubic specimen were coated with Vaseline before exposure, leaving only two opposite exposed surfaces in contact with the sulfate solution. After 60 d of corrosion under the corresponding conditions, the specimens were removed and split along the exposure direction for subsequent layered sampling analysis.

**Figure 4:**
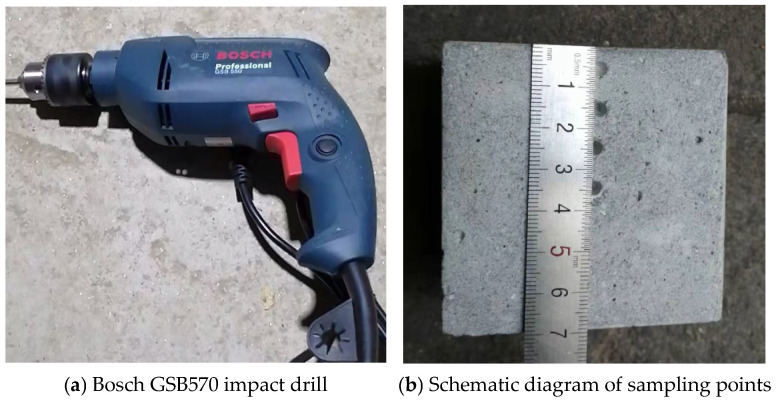
Preparation of samples for sulfate ion content testing.

After splitting the specimens, powder samples were collected by drilling at different depths along the exposure direction using an impact drill, as shown in Figure 4. The sampling depths from the exposed surface were 5 mm, 15 mm, 25 mm, and 35 mm, which were used to characterize the distribution of sulfate ions along the ingress direction. To ensure sampling accuracy, a Bosch GSB570 impact drill and a four-flute 3 mm drill bit (Stuttgart, Germany) were used. The obtained powder samples were labeled and stored in sealed bags. Subsequently, the samples were ground in a mortar and passed through a 200-mesh sieve to remove larger quartz sand particles and improve sample homogeneity.

### 2.5. Testing Methods and Data Processing

#### 2.5.1. Compressive Strength

The compressive strength of mortar specimens was determined in accordance with the Chinese standard JGJ/T 70-2009 (Standard for Test Method of Basic Properties of Construction Mortar) [31]. Tests were conducted using an electro-hydraulic servo testing machine on cube specimens (70.7 mm). The compressive strength was calculated by dividing the failure load by the loaded area and applying a correction factor of 1.35.

Compressive strength tests were conducted for each group at exposure ages of 30 d, 60 d, 90 d, and 120 d. Three replicate specimens were used for each test condition. Error bars in the compressive strength figures represent the dispersion of the three replicate test results. To compare the effects of limestone powder content and water pressure on compressive strength, two-way analysis of variance (two-way ANOVA) was performed using the raw compressive strength data from the three replicate specimens at each exposure age, with the interaction between the two factors included in the model. Statistical significance was accepted at *p* < 0.05, and the effect size was expressed using η_p_^2^ (partial eta squared). All statistical analyses were performed using Python 3.10 with the scipy and statsmodels packages.

#### 2.5.2. Observation of Macroscopic Deterioration Morphology

After reaching the specified exposure ages, the specimen surfaces were cleaned and photographed to record their apparent deterioration characteristics. The observations mainly included changes in surface color, surface roughening, localized peeling, softening, argillization, and corner damage. These observations were used mainly to characterize the evolution of surface damage and to correlate the macroscopic deterioration with changes in compressive strength and microstructural characterization results.

#### 2.5.3. X-Ray Diffraction (XRD) Testing

XRD analysis was performed using a D8 ADVANCE diffractometer (Bruker, Karlsruhe, Germany), with an angular reproducibility of ±0.0001° and a scanning range of 3–105° (2θ). XRD was mainly used to identify the phase composition of mortar corrosion products under different exposure conditions and to analyze the influence of water pressure and limestone powder content on the evolution of the main corrosion phases. Considering that some diffraction peaks of ettringite and thaumasite overlap, the XRD results were used mainly as preliminary evidence for phase identification and were further interpreted in combination with FT-IR and SEM/EDS results.

#### 2.5.4. Fourier-Transform Infrared Spectroscopy (FT-IR) Testing

FT-IR analysis was conducted using a VERTEX 80v spectrometer (Bruker, Karlsruhe, Germany), with a spectral range of 8000–350 cm^−1^ and a resolution of 0.06 cm^−1^. FT-IR was mainly used to identify characteristic functional groups in the corrosion products and, together with XRD results, to support the analysis of the formation characteristics of ettringite, thaumasite, and other related corrosion products.

#### 2.5.5. Scanning Electron Microscopy and Energy-Dispersive Spectroscopy (SEM/EDS)

The microstructure and elemental composition of corrosion products were analyzed using a Quanta 250 scanning electron microscope (FEI, Hillsboro, OR, USA) equipped with an EDS system. SEM was mainly used to observe the micromorphological features of the corrosion products, while EDS was used to obtain the elemental composition of the corresponding regions and thereby support the interpretation of the spatial distribution of different corrosion products. Considering that XRD, FT-IR, and SEM/EDS provide mainly qualitative or semi-quantitative evidence in this study, these results were used to support the analysis of corrosion product types and their distribution characteristics rather than for rigorous quantitative phase composition calculations.

#### 2.5.6. Determination of Soluble SO_4_^2−^ Content

**Figure 5:**
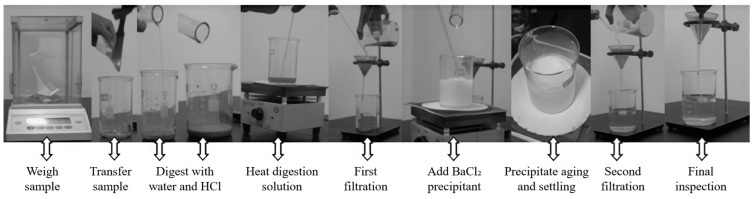
Procedure for soluble SO_4_^2−^ content determination.

The soluble SO_4_^2−^ content was determined in accordance with the Chinese standard GB/T 11899-1989 (Water Quality—Determination of Sulfate—Gravimetric Method) [32]. The specific procedure was as follows. First, the weighed mortar powder was placed in a beaker, and 10 mL of hydrochloric acid and 35 mL of distilled water were added. The mixture was heated to boiling to remove CO_3_^2−^ from the sample and fully dissolve soluble SO_4_^2−^. The slurry was then filtered and washed. The filtrate was made up to 200 mL, and 10 mL of 10 vol% BaCl_2_ solution was added to precipitate SO_4_^2−^ as BaSO_4_. After standing for more than 4 h, the solution was filtered again. During washing, 1 vol% AgNO_3_ solution was used to check whether Cl^−^ remained in the filtrate until no white flocculent precipitate formed. The filter paper containing the precipitate was then placed together with the crucible in a high-temperature furnace for ignition and ashing. After cooling, the mass of the crucible and the total mass of the crucible and precipitate were weighed. Figure 5 shows the procedure for soluble SO_4_^2−^ content determination. The soluble SO_4_^2−^ content in the sample was calculated according to the conversion relationship between BaSO_4_ precipitate mass and SO_4_^2−^.

(1) $$W_{{SO}_{4}^{2-}}=\frac{0.4199\left(m_{2}-m_{1}\right)}{m}\times 100\%$$

Here, *m* is the mass of the weighed powder sample, g; *m*_1_ is the mass of the crucible, g; and *m*_2_ is the total mass of the precipitate and crucible, g. The test results were used to obtain the SO_4_^2−^ content distribution at different depths and to support the interpretation of the influence of water pressure on sulfate ingress characteristics.

## 3. Results

### 3.1. Macroscopic Performance

#### 3.1.1. Compressive Strength Analysis

**Figure 6:**
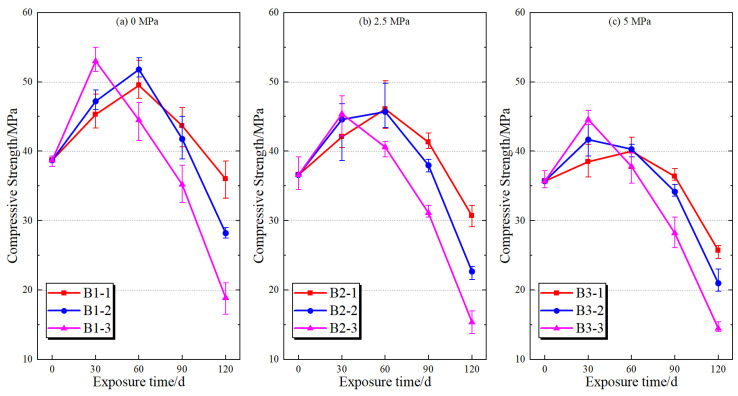
Variation in the compressive strength of mortar specimens with exposure time under different water pressure conditions.

**Figure 7:**
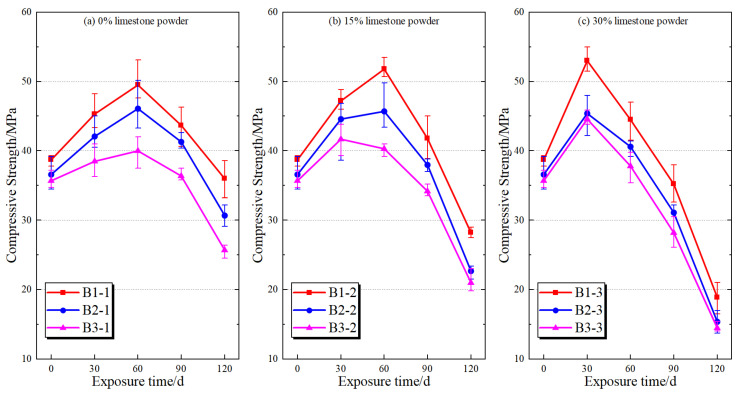
Variation in the compressive strength of mortar specimens with exposure time under different limestone powder contents.

The variation in compressive strength of mortar specimens under different water pressure conditions and limestone powder contents is presented in Figure 6 and Figure 7, respectively. Overall, the compressive strength exhibits a characteristic trend of initial increase followed by a significant decline with increasing exposure time, and this trend becomes more pronounced with increasing water pressure and limestone powder content. The initial strength increase can be attributed to the pore-filling effect of early corrosion products, such as gypsum and ettringite, which temporarily densify the matrix [29,30]. However, this apparent enhancement is transient and does not reflect an actual improvement in durability.

With increasing exposure time, the compressive strength gradually decreases under all test conditions, and the rate of strength loss is clearly influenced by water pressure. Under a water pressure of 0 MPa, the strength degradation remains relatively limited, indicating that sulfate ingress and internal damage development are slower in the absence of water pressure-driven transport. When the water pressure increases to 2.5 MPa, the strength reduction becomes more evident. Under a water pressure of 5.0 MPa, the strength degradation is the most severe, indicating that elevated water pressure accelerates the performance degradation of mortar during sulfate exposure.

The influence of limestone powder content is more prominent under elevated water pressure. Under 5.0 MPa, the strength loss of specimens with 0%, 15%, and 30% limestone powder reach 51.16%, 57.92% and 59.38%, respectively. A consistent trend is observed across all conditions. For a given limestone powder content, compressive strength decreases with increasing water pressure, while for a given water pressure level, strength degradation increases with increasing limestone powder content. These results indicate that water pressure and limestone powder content jointly affect the later-stage performance degradation of mortar. Elevated water pressure provides stronger conditions for sulfate transport, while higher limestone powder content increases the material basis for carbonate-related reactions.

To further quantify the effects of water pressure and limestone powder content on compressive strength, two-way ANOVA was performed separately for each exposure age, and the results are summarized in Table 4. At 30, 60, and 90 d, the main effects of both water pressure and limestone powder content are statistically significant, whereas their interaction is not significant, indicating that the effects of the two factors on strength during the early and middle stages of corrosion exposure are mainly expressed as relatively independent additive effects.

At 120 d, water pressure, limestone powder content, and their interaction all reach statistical significance. The effect of water pressure on compressive strength is significant, F(2,18) = 56.052, *p* < 0.001, η_p_^2^ = 0.862; the effect of limestone powder content is even more pronounced, F(2,18) = 218.055, *p* < 0.001, η_p_^2^ = 0.960. The interaction between water pressure and limestone powder content is also significant, F(4,18) = 3.387, *p* = 0.031, η_p_^2^ = 0.429. These results indicate that the effects of water pressure and limestone powder content on later-stage strength degradation of mortar are not completely independent, but exhibit a certain coupling effect during the later deterioration stage. This statistical result provides macroscopic performance evidence for discussing the coupled effect of these two factors from the perspective of transport and reaction in the following sections.

The simple effect analysis at 120 d further shows that, under different water pressure conditions, limestone powder content significantly affects the final compressive strength of the specimens. The overall order is 0% limestone powder > 15% limestone powder > 30% limestone powder. Under the same limestone powder content, water pressure also affects compressive strength; however, when the limestone powder content reaches 30%, the strength difference among different water pressure groups becomes somewhat reduced. These statistical results are consistent with the measured strength trends and support the interpretation that water pressure and limestone powder content jointly influence later-stage deterioration of mortar.

#### 3.1.2. Macroscopic Deterioration

**Figure 8:**
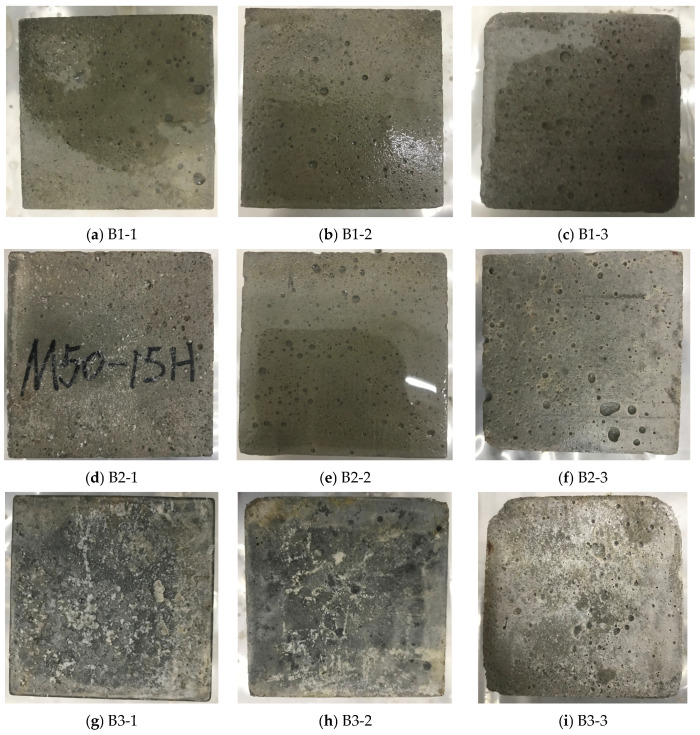
Macroscopic deterioration of mortar specimens after 120 days of exposure under different water pressure conditions and limestone powder contents.

The macroscopic deterioration characteristics of mortar specimens under different water pressure conditions and limestone powder contents are presented in Figure 8. Significant differences in surface morphology are observed with increasing exposure time, water pressure, and limestone powder content. These changes are generally consistent with the evolution of compressive strength, indicating that macroscopic deterioration morphology can reflect the overall trend of material damage development.

Under a water pressure of 0 MPa, the specimens remain relatively intact throughout the exposure period, with only slight surface roughening and limited localized peeling observed. The overall structural integrity is well maintained, which corresponds to the relatively small strength loss observed in Section 3.1.1. When the water pressure increases to 2.5 MPa, the deterioration becomes more noticeable, characterized by surface softening, localized peeling, and the gradual development of loose structures, reflecting the enhanced sulfate ingress and accelerated degradation process.

Under a water pressure of 5.0 MPa, the deterioration becomes significantly more severe. The specimen surfaces exhibit extensive softening, peeling, and the formation of gray–white, mud-like substances, particularly at the edges and corners, where stress concentration and fluid penetration are more pronounced. These regions may be more prone to early damage because of stress concentration and shorter fluid ingress paths. The macroscopic surface argillization corresponds to the significant decrease in compressive strength, indicating consistency between surface damage and overall performance degradation under elevated water pressure.

At the same water pressure level, specimens with higher limestone powder content exhibit earlier onset of deterioration and more severe surface damage. This phenomenon may be related to the carbonate source provided by limestone powder and the associated changes in the pore structure of the matrix, which provide more favorable conditions for the formation of TSA-related products and sulfate ingress. In other words, the macroscopic deterioration morphology also reflects the combined action of water pressure and limestone powder content. Specifically, water pressure enhances the conditions for the aggressive medium to enter the mortar, while limestone powder increases carbonate availability. The two jointly promote the development of surface softening and argillization. It should be noted that macroscopic deterioration observations mainly provide visual evidence of surface damage, and internal crack propagation still needs to be further verified using crack quantification, μCT, or pore structure testing.

#### 3.1.3. Depth-Dependent Distribution of Soluble SO_4_^2−^ Content

**Figure 9:**
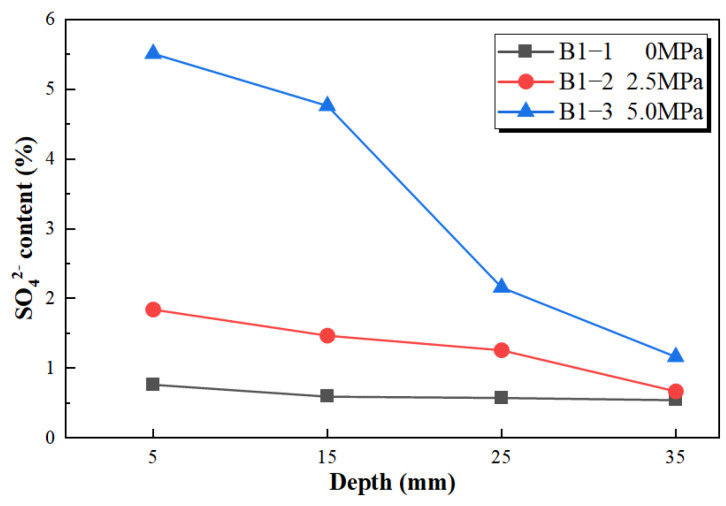
Depth-dependent distribution of soluble SO_4_^2−^ content in B1-series specimens after 60 d of sulfate exposure under different water pressure conditions.

To further illustrate sulfate ingress behavior under different water pressure conditions, the depth-dependent distribution of soluble SO_4_^2−^ content was measured in B1-series specimens without limestone powder after 60 d of corrosion exposure, as shown in Figure 9. Overall, the SO_4_^2−^ content decreases with increasing distance from the exposed surface under all water pressure conditions, indicating that sulfate mainly migrates from the exposed surface toward the interior of the specimen.

The SO_4_^2−^ content distributions differ clearly under different water pressure conditions. Under 0 MPa, the soluble SO_4_^2−^ content at different depths is generally low, with only a certain increase near the surface region. When the water pressure increases to 2.5 MPa, the SO_4_^2−^ content increases both near the surface and within a certain depth range. Under 5.0 MPa, the SO_4_^2−^ content further increases, and relatively high sulfate contents can still be detected at deeper positions.

These results indicate that elevated water pressure helps promote the ingress of sulfate solution into the mortar matrix and expands the depth range affected by sulfate. This provides direct chemical composition evidence for the interpretation that water pressure promotes sulfate transport. Because this test was conducted only on representative B1-series specimens at 60 d, it is used in this paper only as supporting evidence to illustrate the sulfate ingress trend under different water pressure conditions.

### 3.2. Microstructural Characteristics

#### 3.2.1. XRD Analysis

**Figure 10:**
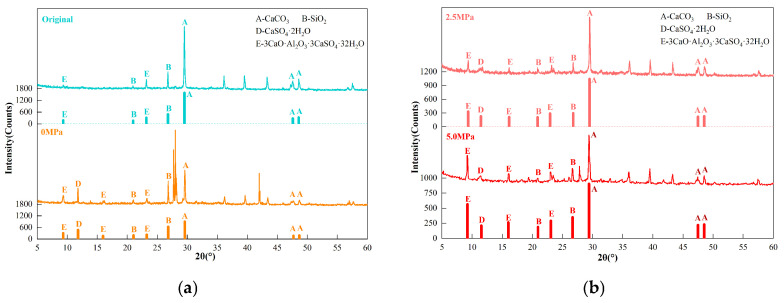
XRD patterns of corrosion products of mortar specimens with 30% limestone powder after 120 days of exposure: (**a**) uncorroded specimen and specimen exposed to 0 MPa; (**b**) specimens exposed to elevated water pressure (2.5 and 5.0 MPa).

Based on the compressive strength results, specimens with a limestone powder content of 30% (B3 series) exhibited the most severe deterioration. Therefore, the gray–white, mud-like corrosion products collected from the specimen surfaces after 120 days of exposure under different water pressure conditions (0, 2.5, and 5.0 MPa) were selected for XRD analysis, as shown in Figure 10. It should be noted that the B3-series samples were used to reveal representative corrosion product characteristics under relatively severe deterioration conditions.

The XRD patterns show that the uncorroded mortar mainly contains calcium carbonate and silica and may also contain peaks of ettringite and thaumasite, which may be attributed to hydration during curing. After sulfate exposure, diffraction peaks related to gypsum, ettringite, and thaumasite are detected under all water pressure conditions, while calcium carbonate and silica mainly originate from the original matrix and residual limestone powder.

A significant influence of water pressure on the evolution of corrosion products is observed. Under 0 MPa, gypsum-related characteristic peaks are relatively evident, indicating that gypsum is one of the important corrosion products under atmospheric immersion. Under 2.5 MPa, peaks related to gypsum, ettringite, and thaumasite appear simultaneously, suggesting a more complex corrosion product assemblage. When the water pressure increases to 5.0 MPa, the intensities of peaks related to ettringite/thaumasite near approximately 9° and 15° (2θ) increase, whereas the gypsum peaks become relatively weaker. This suggests that elevated water pressure may promote further participation of sulfate in aluminate- and silicate-related reactions.

It should be noted that, due to the similar crystal structures of ettringite and thaumasite, some of their diffraction peaks overlap, making it difficult to distinguish these two phases solely by XRD. Therefore, the XRD results are not used in this study as the sole basis for identifying pure ettringite or thaumasite phases, but are interpreted comprehensively in combination with FT-IR and SEM/EDS results.

#### 3.2.2. FT-IR Analysis

**Figure 11:**
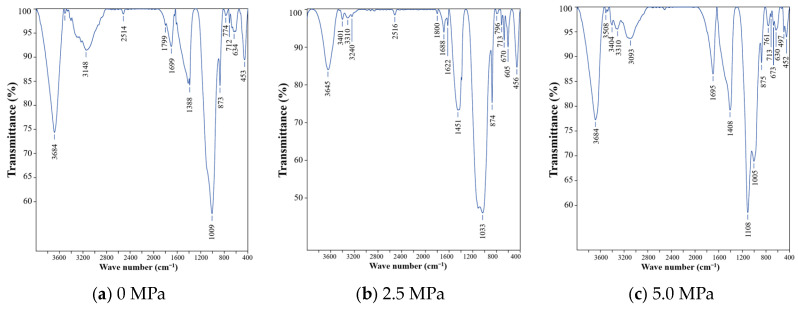
FT-IR spectra of surface corrosion products after 120 days of exposure: (**a**) 0 MPa; (**b**) 2.5 MPa; (**c**) 5.0 MPa.

To further identify the composition of the gray–white, mud-like corrosion products and distinguish between ettringite and thaumasite, FT-IR analysis was conducted on the surface materials of specimens exposed to different water pressure conditions for 120 d, as shown in Figure 11. The characteristic wavenumbers of relevant functional groups are summarized in Table 5.

The FT-IR spectra reveal that the intensity of the sulfate-related absorption band (around 1100 cm^−1^) increases significantly with increasing water pressure. Under 5.0 MPa, a relatively pronounced absorption peak is observed at approximately 1108 cm^−1^, whereas this peak is weak under 0 MPa. This result indicates that the content of sulfate-related components in the corrosion products is higher under elevated water pressure, which is consistent with the XRD results.

In addition, the absorption bands near approximately 497 cm^−1^, 669–713 cm^−1^, and 875 cm^−1^ can be associated with [SiO_6_] octahedral units and CO_3_^2−^ groups, separately, and these characteristics are consistent with the structural features of thaumasite. Meanwhile, the absorption peak related to [AlO_6_] near approximately 850 cm^−1^ is not obvious, suggesting that the gray–white, mud-like surface products are more consistent with thaumasite-rich assemblages rather than being solely controlled by ettringite.

Therefore, the FT-IR results support the following interpretation: under elevated water pressure and high limestone powder content, the surface corrosion products exhibit evident carbonate-, sulfate-, and silicate-related features and are consistent with thaumasite-rich products. This result also indicates that sulfate supply promoted by water pressure and carbonate supply provided by limestone powder may jointly change the formation characteristics of surface corrosion products.

#### 3.2.3. SEM/EDS Analysis

To further elucidate the microstructural characteristics and chemical composition of the corrosion products, SEM and EDS analyses were conducted on both the surface and internal regions of specimens with 30% limestone powder (B3 series) after 120 days of exposure under 5.0 MPa water pressure. This sample represents a relatively severe deterioration condition in the present test and was therefore used mainly to illustrate the typical morphology and spatial distribution characteristics of corrosion products.

**Figure 12:**
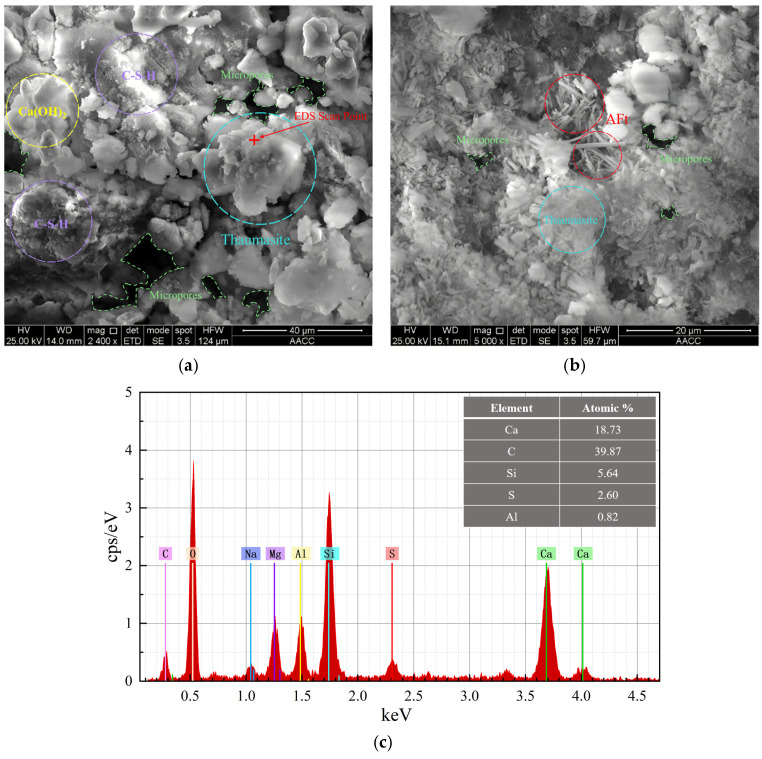
SEM images and EDS analysis of surface corrosion products: (**a**,**b**) SEM images; (**c**) EDS spectrum. Yellow dashed circle: Ca(OH)_2_; purple dashed circles: C-S-H gel; blue dashed circle: thaumasite; red dashed circles: AFt; green dashed outlines: micropores.

The SEM images of the specimen surface (Figure 12a,b) show that the corrosion products exhibit a loose, porous, and mud-like morphology, with no well-defined crystalline structure. This morphology is consistent with the typical features of thaumasite, which lacks cementitious properties and contributes to surface softening and argillization. The corresponding EDS result (Figure 12c) indicates that the surface products are mainly composed of Ca, C, Si, S, and O elements, showing that sulfate-, carbonate- and silicate-related components are simultaneously enriched in this region. Considering that EDS atomic ratios only reflect the elemental composition of the tested micro-region and can be affected by residual matrix, mixed hydration products, and the selection of the testing area, this study makes a comprehensive interpretation by combining SEM morphology, EDS elemental composition, FT-IR characteristic bands, and XRD results. Accordingly, the gray–white, mud-like surface corrosion products are considered to be consistent with thaumasite-rich assemblages.

**Figure 13:**
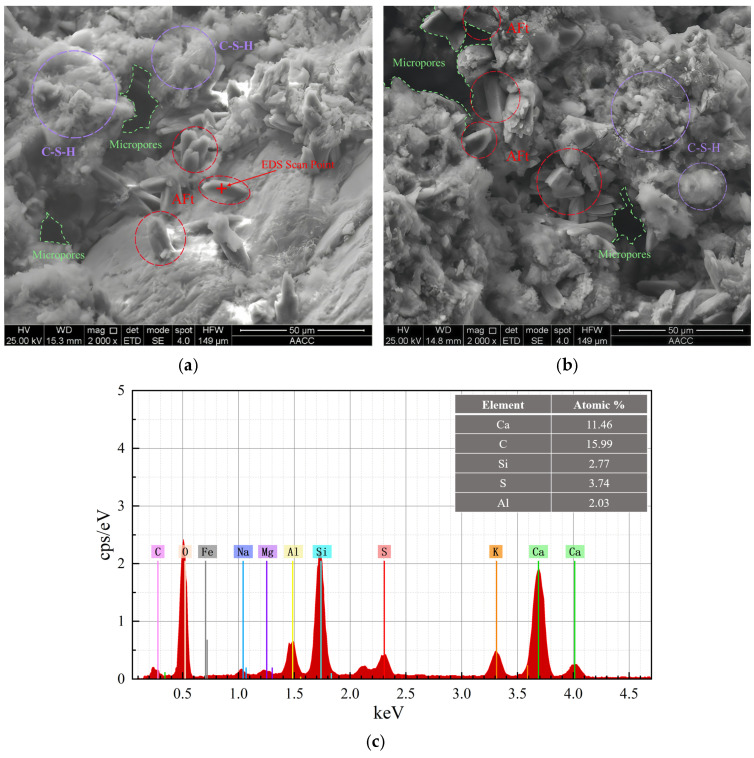
SEM images and EDS analysis of internal corrosion products: (**a**,**b**) SEM images; (**c**) EDS spectrum.

In contrast, the internal corrosion products show clearly different microstructural characteristics. As shown in Figure 13a,b, numerous columnar and needle-like crystals are observed in the internal region. These crystals fill the pores and may induce local expansive stress during continued growth. The EDS analysis (Figure 13c) shows that these products are mainly composed of Ca, Al, S, and O elements, with Al/S and Al/Ca atomic ratios of approximately 1:1.87 and 1:5.73, respectively, which are consistent with the stoichiometric characteristics of ettringite. These results, together with the observed crystal morphology, indicate that the internal corrosion products are consistent with ettringite-rich assemblages.

Based on the morphology and elemental composition, a certain spatial differentiation of surface and internal corrosion products can be inferred under elevated water pressure. The surface directly contacts the external sulfate-rich solution, and limestone powder increases local carbonate availability; therefore, thaumasite-rich products are more likely to form at the surface. In the internal region, carbonate availability is relatively limited, and the reaction environment differs from that at the surface. Therefore, ettringite-rich products are more likely to form. This spatial differentiation provides microstructural support for the interpretation that water pressure-driven transport and carbonate supply jointly affect the distribution of TSA-related products. It should be emphasized that this interpretation of spatial differentiation is based on representative SEM/EDS regions and spectral results, and full cross-sectional quantitative mapping or quantitative phase analysis was not performed. Therefore, it should be regarded as a qualitative interpretation rather than a strict quantitative conclusion.

## 4. Discussion

The above results on macroscopic performance, depth-dependent soluble SO_4_^2−^ content distribution, and microstructural characteristics indicate that water pressure and limestone powder content jointly affect the deterioration process of cement mortar in a sulfate environment. To avoid overinterpreting the experimental results as direct proof of the mechanism, this section discusses the internal relationships among the experimental observations from the perspectives of water pressure, limestone powder, their coupled effect, environmental interactions, and study limitations.

### 4.1. Effect of Water Pressure on Sulfate Ingress and Damage Development

Water pressure is an important external factor affecting sulfate ingress and the deterioration rate of mortar. Compared with atmospheric immersion, elevated water pressure can increase the likelihood that the external sulfate solution enters pores, interfacial transition zones, and pre-existing microdefects, thereby increasing the opportunity for sulfate ions to contact and react with cement hydration products. In this study, the depth-dependent soluble SO_4_^2−^ content distribution of the B1-series specimens shows that the SO_4_^2−^ content at different depths inside the specimens generally increases with increasing water pressure, providing direct chemical evidence that water pressure promotes sulfate ingress. This interpretation is generally consistent with recent studies that describe external sulfate attack as a coupled process involving ion transport, chemical reactions, and damage development [4].

The compressive strength results also show that higher water pressure leads to more pronounced later-stage strength loss. Under elevated water pressure, early corrosion products may fill part of the pore space, resulting in a temporary increase in strength. However, as exposure proceeds, the continuous formation of corrosion products and the crystallization stress induced by them gradually exceed the beneficial effect of pore filling, leading to strength loss and aggravated surface damage.

It should be noted that crack width, crack number, porosity, permeability coefficient, and water absorption were not directly measured in this study. Therefore, the discussion on the role of water pressure in promoting sulfate ingress and damage development should be understood as a mechanistic interpretation based on the existing macroscopic performance, apparent morphology, and SO_4_^2−^ content distribution results, and it still needs to be verified with further pore structure and permeability tests. Future studies may use μCT, MIP, crack image analysis, and permeability testing to further quantify the evolution of the pore–crack structure under water pressure.

### 4.2. Effect of Limestone Powder on TSA Deterioration

The influence of limestone powder on TSA deterioration is mainly reflected in two aspects. First, limestone powder can provide a carbonate source for the system, and carbonate is one of the important components required for thaumasite formation [26,28]. In this study, with increasing limestone powder content, the surface argillization of the specimens becomes more obvious, and the FT-IR characteristic absorptions related to CO_3_^2−^ and [SiO_6_] structures become more pronounced. This indicates that limestone powder increases the possibility of forming thaumasite-rich products. This interpretation is consistent with recent studies indicating that limestone powder may increase the susceptibility of cementitious materials to thaumasite-related deterioration by providing carbonate availability under favorable sulfate exposure conditions [26,33].

Second, limestone powder, as a low-reactivity or inert filler, may alter the pore structure and interfacial characteristics of the mortar matrix. Previous studies have shown that a higher limestone powder content may increase the proportion of capillary pores and connected pores, thereby affecting ion migration and permeation behavior [26,34,35]. Under elevated water pressure, such structural characteristics may further amplify the ingress of sulfate solution into the mortar matrix and thus intensify later-stage strength loss.

It should be noted that pore structure parameters were not directly measured in this study. Therefore, the discussion on limestone powder-induced changes in pore connectivity is mainly based on the existing literature and the integrated interpretation of strength, morphology, and microstructural product changes observed in this test. To further clarify the influence of limestone powder on transport pathways, future work may combine MIP, low-field nuclear magnetic resonance, water absorption, and permeability tests for verification.

### 4.3. Coupled Effect of Water Pressure and Limestone Powder

The results of this study indicate that water pressure and limestone powder do not affect TSA deterioration in isolation, but act jointly at both the transport and reaction levels. From the transport perspective, elevated water pressure promotes the ingress of sulfate solution into mortar. From the reaction perspective, limestone powder increases carbonate availability and provides material conditions for the formation of thaumasite-related products. When the two factors are present simultaneously, sulfate supply and carbonate supply are more likely to be coupled in the surface region, thereby accelerating surface softening and argillization. The two-way ANOVA results show that, at an exposure age of 120 d, the interaction between water pressure and limestone powder content has a significant effect on compressive strength, supporting from the macroscopic performance perspective that the two factors jointly affect later-stage deterioration of mortar. Based on this, the coupled effect is further interpreted through a pathway of “transport enhancement–carbonate supply–corrosion product evolution–aggravated macroscopic damage”. This interpretation is consistent with recent reviews describing sulfate deterioration as a coupled transport–reaction–damage process, while the present study further highlights the combined role of water pressure-assisted sulfate supply and limestone-powder-derived carbonate availability [4,5].

Recent studies conducted under atmospheric or low-pressure sulfate exposure conditions have shown that TSA in carbonate-bearing cementitious materials is strongly governed by temperature, moisture availability, and carbonate supply. Wang et al. reported that, under low-temperature sulfate exposure, mortars containing limestone powder suffered TSA-related deterioration after long-term exposure, with strength losses of 17.4–48.7% after 360 d, whereas thaumasite was not detected in specimens without limestone powder [33]. Beltrame et al. also found that thaumasite formation in Portland limestone cement mortars was more pronounced under continuous immersion than under wetting/drying cycles, indicating that sustained moisture supply and ion transfer are important for TSA development [36]. In addition, Song and Ma observed long-term TSA in carbonate-bearing mortars exposed to Na_2_SO_4_ solution at low temperature, further confirming the sensitivity of TSA to carbonate availability and exposure conditions [29]. Compared with these atmospheric or low-pressure studies, the present results indicate that elevated water pressure may further accelerate TSA deterioration by promoting sulfate ingress and expanding the affected depth. Therefore, the coupled deterioration observed in this study is not only associated with limestone powder-derived carbonate availability, but also with water pressure-assisted sulfate transport, which distinguishes it from conventional immersion-based TSA deterioration.

From the perspective of possible transport pathways, the incorporation of limestone powder increases the carbonate source in the system and may also change the pore structure and connectivity of the mortar matrix. Since limestone powder mainly exists as filler particles, partial replacement of cement reduces the cement clinker content and the amount of hydration products formed, which may decrease matrix compactness and provide more favorable pathways for external sulfate solution to enter the mortar. Under elevated water pressure, the external sulfate solution is more likely to migrate inward along surface pores, interfacial transition zones, and pre-existing defects, thereby increasing the contact opportunity between sulfate and hydration products. This interpretation is consistent with the soluble SO_4_^2−^ content test results, which show that the SO_4_^2−^ content at different depths inside the mortar generally increases with increasing water pressure, indicating that water pressure promotes sulfate ingress.

From the perspective of reaction conditions, the carbonate source provided by limestone powder and the continuous sulfate supply promoted by water pressure jointly create a favorable environment for the formation of thaumasite-related products. Thaumasite formation requires the participation of sulfate, carbonate, calcium, and silicon sources. Therefore, sulfate ingress alone or carbonate supply alone is insufficient to fully explain the severe surface argillization observed under conditions of elevated water pressure and high limestone powder content. Under the present test conditions, elevated water pressure may enhance the migration ability of SO_4_^2−^ within the mortar, while higher limestone powder content increases the availability of CO_3_^2−^. The combined action of these two factors makes the formation of thaumasite-rich corrosion product assemblages more likely in the surface region.

This coupled effect is ultimately manifested as aggravated macroscopic deterioration. Early corrosion products may fill part of the pores, causing a temporary increase in compressive strength. However, as exposure time increases, sulfate reaction products continue to form. Thaumasite-rich, mud-like products at the surface weaken the cementing ability of the matrix, while ettringite-rich products in the interior may generate expansive stress and induce structural damage. Therefore, under the combined action of elevated water pressure and high limestone powder content, mortar exhibits more severe surface softening, peeling, and strength loss. In other words, limestone powder mainly provides the material basis for TSA development, whereas water pressure mainly enhances the ingress of aggressive media and the reaction conditions. The two factors jointly amplify the deterioration process of mortar.

Therefore, this study suggests that water pressure and limestone powder do not simply affect TSA independently, but may form a coupled effect through a pathway of “transport enhancement–carbonate supply–corrosion product evolution–aggravated macroscopic damage”. Nevertheless, this mechanism remains a mechanistic interpretation based on the existing macroscopic performance, SO_4_^2−^ content distribution, and microstructural characterization results, and further verification using pore structure testing, permeability testing, and quantitative phase analysis is still required.

### 4.4. Environmental Interactions and Engineering Implications

In real deep underground environments, sulfate attack is usually controlled by multiple environmental factors, including sulfate concentration, water pressure, temperature, groundwater renewal rate, ion supply from surrounding rock or soil, pH conditions, and long-term loading state. In this study, a 10 wt% Na_2_SO_4_ solution, a low-temperature exposure environment of approximately (12 ± 5) °C, and water pressures of 0–5.0 MPa were used mainly to highlight the effects of water pressure and limestone powder content on TSA deterioration.

From an engineering perspective, deep shaft lining concrete is usually exposed for a long time to sulfate-rich groundwater and elevated water pressure. If the material system contains a relatively high carbonate source, the risk of TSA-related deterioration may increase. The results of this study suggest that, in elevated water pressure sulfate environments, attention should be paid to the coupling among material composition, pore structure, external water pressure, and ion supply, rather than inferring the service behavior of deep engineering structures solely from sulfate attack mechanisms obtained under atmospheric immersion conditions. For cement-based materials containing limestone powder or a high carbonate source, the risk of transport–reaction coupling under elevated water pressure should be given particular attention.

However, real groundwater is usually a multi-ion system, and temperature, pressure, and flow conditions vary with time and space. The present test used a single Na_2_SO_4_ solution and controlled water pressure conditions, which cannot fully represent actual service environments. Therefore, future studies should further consider the combined action of multiple ions such as Mg^2+^, Cl^−^, and HCO_3_^−^, as well as the effects of temperature fluctuation, long-term seepage, and stress coupling on the TSA deterioration process.

### 4.5. Study Limitations

This study analyzed TSA deterioration under the coupled effects of elevated water pressure and limestone powder using compressive strength, macroscopic morphology, depth-dependent soluble SO_4_^2−^ content distribution, XRD, FT-IR, and SEM/EDS results. To clarify the scope of applicability of the conclusions, the following limitations should be noted.

First, the soluble SO_4_^2−^ content test was conducted only on representative B1-series specimens at 60 d and was mainly used to support the interpretation of the influence of water pressure on sulfate ingress. It cannot be regarded as a complete quantitative description of sulfate transport behavior for all mixture proportions and all exposure ages.

Second, XRD, FT-IR, and SEM/EDS analyses were mainly conducted on representative samples. In particular, SEM/EDS analysis was mainly performed on representative surface and internal regions of B3-series specimens exposed to 5.0 MPa for 120 d, which showed relatively severe deterioration. Therefore, the interpretation of thaumasite-rich surface assemblages and ettringite-rich internal assemblages should be understood as a comprehensive qualitative result based on multiple characterization methods under representative conditions and should not be directly generalized as a quantitative rule for all test groups.

Finally, MIP, μCT, quantitative crack width analysis, permeability or water absorption tests, and Rietveld quantitative phase analysis were not conducted in this study. Therefore, the discussion on pore structure changes and the transport–damage coupling process remains a mechanistic interpretation. Future studies may further combine pore-structure characterization, seepage properties, and quantitative phase analysis to improve the understanding of the transport–reaction–damage mechanism of TSA under elevated water pressure.

## 5. Conclusions

The following conclusions can be drawn:

(1) Elevated water pressure aggravates the macroscopic deterioration of mortar under sulfate exposure. The soluble SO_4_^2−^ content distribution of B1-series specimens at 60 d shows that both the SO_4_^2−^ content inside the mortar and the affected depth increase with increasing water pressure, indicating that water pressure promotes the migration of sulfate solution into the mortar.
(2) The compressive strength of mortar exhibits a trend of initial increase followed by decrease with increasing exposure time. The early strength increase may be related to pore filling by corrosion products, whereas the later strength decrease is associated with the continuous formation of corrosion products, accumulation of crystallization stress, and development of microstructural damage. Under 5.0 MPa, the strength losses of specimens with 0%, 15%, and 30% limestone powder contents reach 51.16%, 57.92%, and 59.38%, respectively.
(3) Two-way ANOVA shows that both water pressure and limestone powder content have significant effects on compressive strength. At 120 d, their interaction reaches statistical significance, indicating that the influence of limestone powder content on later-stage strength loss has a certain coupling effect with water pressure level.
(4) The combined XRD, FT-IR, and SEM/EDS results indicate that, with increasing water pressure, the corrosion products gradually evolve from being dominated by gypsum-related products to ettringite- and thaumasite-related products. Under high limestone powder content and 5.0 MPa water pressure, the corrosion products show a certain spatial differentiation: the gray–white, mud-like surface products are consistent with thaumasite-rich assemblages, whereas the needle- and column-like crystals in the interior are consistent with ettringite-rich assemblages.
(5) Overall, elevated water pressure promotes sulfate transport, while limestone powder increases carbonate availability. These two factors may jointly intensify TSA deterioration of mortar through a pathway of “transport enhancement–carbonate supply–corrosion product evolution–aggravated macroscopic damage”. This result indicates that, in deep underground sulfate environments under elevated water pressure, durability assessment of limestone powder-containing cement-based materials should focus on the coupling between water pressure-driven transport and carbonate supply.

## Figures and Tables

**Table 1:** Chemical composition of cement.

Composition	SO_3_	Na_2_O	Fe_2_O_3_	MgO	Al_2_O_3_	CO_3_	CaO	SiO_2_
Proportion/%	1.94	0.89	2.36	1.32	6.94	1.42	61.27	21.04

**Table 2:** Mix proportions of mortar with different limestone powder contents.

Mix ID	Cement (kg/m^3^)	Limestone Powder (kg/m^3^)	Sand (kg/m^3^)	Water (kg/m^3^)	Water-to-Binder Ratio
B1 (M50C)	450	0	1350	225	0.5
B2 (M50-15L)	382.5	67.5	1350	225	0.5
B3 (M50-30L)	315	135	1350	225	0.5

**Table 3:** Experimental design and grouping of mortar specimens under different limestone powder contents and water pressure conditions.

Group ID	Limestone Powder Content (%)	Water Pressure (MPa)	Na_2_SO_4_ Solution Concentration (wt%)	Exposure Time (d)
B1-1	0	0	10%	30, 60, 90, 120
B1-2	0	2.5	10%	30, 60, 90, 120
B1-3	0	5.0	10%	30, 60, 90, 120
B2-1	15	0	10%	30, 60, 90, 120
B2-2	15	2.5	10%	30, 60, 90, 120
B2-3	15	5.0	10%	30, 60, 90, 120
B3-1	30	0	10%	30, 60, 90, 120
B3-2	30	2.5	10%	30, 60, 90, 120
B3-3	30	5.0	10%	30, 60, 90, 120

**Table 4:** Results of two-way ANOVA for compressive strength at different exposure ages.

Exposure Time (d)	Source	F Value	*p* Value	η_p_^2^
30	Water pressure	18.715	<0.001	0.675
30	Limestone powder content	11.565	<0.001	0.562
30	Water pressure × limestone powder	0.739	0.578	0.141
60	Water pressure	29.913	<0.001	0.769
60	Limestone powder content	9.977	0.001	0.526
60	Water pressure × limestone powder	0.861	0.506	0.161
90	Water pressure	30.588	<0.001	0.773
90	Limestone powder content	49.845	<0.001	0.847
90	Water pressure × limestone powder	0.285	0.884	0.06
120	Water pressure	56.052	<0.001	0.862
120	Limestone powder content	218.055	<0.001	0.96
120	Water pressure × limestone powder	3.387	0.031	0.429

**Table 5:** Characteristic wave number of main chemical bonds in FT-IR diagram.

Chemical Bond	O-H	C-O(CO_3_^2−^)	S-O(SO_4_^2−^)	SiO_4_	SiO_6_	AlO_6_
Wave number/cm^−1^	3200~3600	875, 1400	1100	920~940	500, 669, 710	850

## Data Availability

The data presented in this study are available on request from the corresponding author due to (the data presented in this study were obtained for one industry-collaborative project on coal mine shafts and are classified as confidential information of national significance).
